# Nest covering in plovers: How modifying the visual environment influences egg camouflage

**DOI:** 10.1002/ece3.2494

**Published:** 2016-09-29

**Authors:** Jolyon Troscianko, Jared Wilson‐Aggarwal, Claire N. Spottiswoode, Martin Stevens

**Affiliations:** ^1^ Centre for Ecology and Conservation University of Exeter Penryn UK; ^2^ Department of Zoology University of Cambridge Cambridge UK; ^3^ DST‐NRF Centre of Excellence at the FitzPatrick Institute University of Cape Town Cape Town South Africa

**Keywords:** adaptive camouflage, animal vision, background matching, background modification, crypsis, disruptive coloration, predator vision, shorebird ecology, visual ecology

## Abstract

Camouflage is one of the most widespread antipredator defences, and its mechanistic basis has attracted considerable interest in recent years. The effectiveness of camouflage depends on the interaction between an animal's appearance and its background. Concealment can therefore be improved by changes to an animal's own appearance, by behaviorally selecting an optimal background, or by modifying the background to better match the animal's own appearance. Research to date has largely focussed on the first of these mechanisms, whereas there has been little work on the second and almost none on the third. Even though a number of animal species may potentially modify their environment to improve individual‐specific camouflage, this has rarely if ever been quantitatively investigated, or its adaptive value tested. Kittlitz's plovers (Charadrius pecuarius) use material (stones and vegetation) to cover their nests when predators approach, providing concealment that is independent of the inflexible appearance of the adult or eggs, and that can be adjusted to suit the local surrounding background. We used digital imaging and predator vision modeling to investigate the camouflage properties of covered nests, and whether their camouflage affected their survival. The plovers' nest‐covering materials were consistent with a trade‐off between selecting materials that matched the color of the eggs, while resulting in poorer nest pattern and contrast matching to the nest surroundings. Alternatively, the systematic use of materials with high‐contrast and small‐pattern grain sizes could reflect a deliberate disruptive coloration strategy, whereby high‐contrast material breaks up the telltale outline of the clutch. No camouflage variables predicted nest survival. Our study highlights the potential for camouflage to be enhanced by background modification. This provides a flexible system for modifying an animal's conspicuousness, to which the main limitation may be the available materials rather than the animal's appearance.

## 1 Introduction

Camouflage is a key mechanism for evading predators and offers striking and intuitive examples of natural selection (Wallace, 1867). Considerable research over recent years has focussed on the mechanistic basis of camouflage, typically using artificially made or manipulated stimuli (Cuthill et al., 2005; Schaefer & Stobbe, 2006; Webster, Hassall, Herdman, Godin, & Sherratt, 2013), or laboratory experiments (Chiao, Chubb, Buresch, Siemann, & Hanlon, 2009; Kang, Stevens, Moon, Lee, & Jablonski, 2014b; Lovell, Ruxton, Langridge, & Spencer, 2013; Merilaita & Dimitrova, 2014; Skelhorn, Rowland, Speed, & Ruxton, 2010). However, comparatively few studies have investigated camouflage in natural systems, especially while accounting for predator vision, and even fewer have investigated the ability of animals to select or modify backgrounds to complement their camouflage (e.g., Marshall, Philpot, & Stevens, 2016). The effectiveness of any individual's camouflage depends on the interaction between an animal's appearance and its background (Endler, 1978; Troscianko, Wilson‐Aggarwal, Stevens, & Spottiswoode, 2016). As such, behavioral choice of suitable microhabitats or modification of the visual environment, as opposed to adaptation of the animal's own phenotypic appearance, are additional routes to effective camouflage. Animals that modify their surroundings provide interestingly flexible systems for investigating camouflage in natural systems.

Background selection to enhance an animal's camouflage was a strategy noted by Wallace (1867), who reported that *Kallima* butterflies would only settle on twigs and branches that facilitated the butterfly's concealment. Similar effects have been noted more recently in other species such as coral reef flounders *Bothus lunatus* (Tyrie, Hanlon, Siemann, & Uyarra, 2015), while moths have been shown to use multisensory cues (including vision) when selecting resting positions (Kang, Moon, Lee, & Jablonski, 2013, 2014a), and laboratory experiments on Japanese quail *Coturnix japonica* demonstrated that females with darker eggs chose to nest on darker backgrounds (Lovell et al., 2013). One recent field study of microhabitat choice in reptiles showed that individual Aegean wall lizards (*Podarcis erhardii*) were more likely to be found resting on backgrounds that improved their own individual camouflage to predator vision than on backgrounds that did not (Marshall et al., 2016). Furthermore, this tendency was more pronounced in females living in habitats with higher predation risk, suggesting concealment by habitat selection is dependent on predation pressure. However, beyond the above examples, few studies have tested microhabitat selection for camouflage, especially in the field and with respect to predator vision.

Modification of backgrounds in order to enhance camouflage is a distinct strategy from background selection and a further potential route to concealment. Numerous species decorate their own bodies with elements of the background to conceal themselves (Ruxton & Stevens, 2015), such as blue‐footed boobys *Sula nebouxii* that cake their eggs with mud (Mayani‐Parás, Kilner, Stoddard, Rodríguez, & Drummond, 2015), caddis fly and bagworm moth larvae that construct cases around their bodies, and crustacea that attach sediment (Lee, Parra‐Velandia, Ng, & Todd, 2014) or seaweed to their carapaces (Hultgren & Stachowicz, 2009), thus changing their own appearance. However, background modification refers instead to cases where animals change the appearance of their surroundings. Such an approach is widely reported to be used by some bird species to conceal their nests and eggs (Bailey, Muth, Morgan, Meddle, & Healy, 2014; Hansell, 1996), and also by invertebrates, such as wasps that use lichen and other materials to conceal their nests (Strassmann, Hughes, & Queller, 1990). The structures created by these species often have numerous functions that can be difficult to disentangle from camouflage alone, particularly in nests that hold the eggs off the ground and therefore also serve a structural function. Kittlitz's plovers *Charadrius pecuarius* provide an ideal study system for investigating the camouflage strategies used to conceal nests because the raw materials providing color and pattern matching are not limited by the adults' or the eggs' phenotypic appearance. Furthermore, their nests provide little or no structural or mechanical protection to the eggs, which simply lie within a shallow scrape in the ground, although covering may confer some thermal protection. When leaving their nests, Kittlitz's plovers cover their clutch with plant or inorganic material in a so‐called leaving‐scuffle (Hall, 1958, 1960), standing with their legs either side of the nest and kicking material in from the sides, often rotating on the spot to cover the eggs from all sides (see accompanying video). Nest covering is a concealment strategy found in numerous other bird species, including other Charadriformes (Amat, Monsa, & Masero, 2012; Hall, 1960; Maclean, 1974; Summers & Hockey, 1981) as well as the Anatidae (Fast, Gilchrist, & Clark, 2010; Kreisinger & Albrecht, 2008; Opermanis, 2004) and Podicipedidae (*Tachybaptus*; Prokop & Trnka, 2010).

In addition to camouflage, nest materials also affect the thermal properties and humidity of the nest (Hilton et al., 2004; Prokop & Trnka, 2010), and some Charadriform species may select nest materials that reflect light to protect them from overheating (Mayer et al., 2009), or covering materials that reduce heat loss (Reid et al., 2002). Leaving the nest covered by material from the environment could be advantageous for adult birds, freeing them from incubation so that they can forage, while potentially providing better camouflage than provided by their own bodies. For the Kentish plover *Charadrius alexandrinus*, covering appears to perform a dual function, providing both concealment and thermoregulation (Amat et al., 2012). Amat et al. (2012) found that Kentish plovers left their nests covered most frequently around mid‐morning, a time of day that also created the optimal temperatures for egg development (embryogenesis) in covered nests. Temperatures in our study area (the Western Cape Province of South Africa) during the Kittlitz's plover breeding season are unlikely to overheat the eggs (the August to October time‐frame average high temperatures at the nearby Langebaanweg weather station are 17°–18°, with average low temperatures of 10°–11°, data from 2000 to 2012; World Weather Online 2016). However, covering could help to insulate the eggs from the less damaging lower temperatures caused by strong winds and sometimes heavy rain, which at worst would slow down embryonic development. Nevertheless, concealment has been assumed to be the primary factor driving nest covering in Kittlitz's plovers (Maclean, 1974), and recent data from our study system at the same site demonstrate that uncovered artificial nests designed to mimic Kittlitz's plover nests were more likely to be depredated than covered nests (Ferguson, 2016).

We aimed to determine how the nest‐covering behavior of Kittlitz's plovers affected the appearance of their nests, using measures of camouflage that take into account their main predators' visual systems. If Kittlitz's plovers cover their nests in order to achieve perfect background matching, then we should find no consistent difference between the colors, patterns, and other appearance attributes of the nests and those of their local surroundings. We tested this by measuring the appearance of the nests at a number of distances, creating concentric zones to take into account the gradually changing boundaries of their nests. However, nests are not always fully covered, so we might instead expect the plovers to select materials that complement the eggs themselves. If so, then we would expect the nest material to be a better match to the color or patterns of the eggs than the nest's surroundings or represent a compromise between the two (i.e., a poor match to both that is an intermediate between them). However, limitations in the available materials could force plovers to select materials that match one appearance attribute over another, resulting in a good color or pattern match to the eggs or surrounds, while simultaneously worsening the nests' color or pattern match in other respects. Alternatively, any observed deviations from perfect background matching or egg matching could indicate the use of specific camouflage strategies, such as disruptive coloration whereby high‐contrast nesting material could break up the edges of the clutch (Cuthill et al., 2005).

Here, we investigate the visual characteristics of Kittlitz's plover nests to determine which of these scenarios best describes their nest camouflage strategies. Recently, in a similar system, we found that pattern and contrast matching were most important in the survival of Zambian ground‐nesting birds (Troscianko et al., 2016). Therefore, we might expect the Kittlitz's plovers to focus on matching the pattern of the eggs or backgrounds to reduce the likelihood of predation, although previous studies (that did not take predator vision into account) suggest color could also be valuable for survival (Solis & De Lope, 1995) and as could matching the size of other objects in the local environment (Castilla, Dhondt, Díaz‐Uriarte, & Westmoreland, 2007; Colwell et al., 2011). Finally, we tested whether egg appearance and nest modifications predicted the likelihood of nest predation.

## 2 Methods

### 2.1 Study site and natural history

Kittlitz' plover nests were located in the Western Cape Province of South Africa, around commercial salt pans on the Berg River estuary (Kliphoek Farm, centered on −32.794907, 18.159825; and Cerebos Saltpans, centered on −32.824639, 18.200353), and on the coast at a site between Laingville and the mouth of the Berg River, centered on −32.778601, 18.087064. All sites were on private land, and permission was granted from the landowners and CapeNature. The nature of the salt pan embankments and coastal habitat restricted nest sites to bands of land between the water and either road, tracks, or agricultural land. Nests were located by walking slowly along the potential nesting habitats with binoculars, searching for plovers fleeing their nests and/or performing the leaving‐scuffle behavior, or by exhaustive visual searching during slow systematic patrols. We suspect that the Kittlitz's plovers covered their nests whenever they left them irrespective of the presence of potential threats because we rarely found uncovered nests, even when observing and approaching vacant nests from distances beyond their normal fleeing distances. However, in these cases, we cannot rule out nest covering as a response to prior threats that we did not see. Nests were photographed and their GPS positions logged. Each nest was checked on every second day for evidence of hatching or predation. Motion‐triggered video cameras were placed at a subset of nests to record any predation events.

Camera traps used at our study recorded nest predation by pied crows *Corvus albus* on three occasions, and one additional nest was lost shortly after a pied crow was caught on camera being mobbed by Hartlaub's gulls. One nest disappeared shortly after an African sacred ibis *Threskiornis aethiopicus* was caught on camera, suggesting this could have been the predator if it took the eggs before the motion‐triggered camera could restart filming. Blacksmith plovers nesting near the Kittlitz's plover nests were recorded piercing the eggs of Kittlitz's plovers on two separate occasions; blacksmith plovers pierced one egg at a time, and each pierced egg was later removed by the Kittlitz's plovers. Although the blacksmith plovers were likely not preying upon the nests as food, the survival of Kittlitz's plover nests partially depended on being undetected by blacksmith plovers. Two additional nests that were not monitored by cameras lost one egg, followed by the second a short time later. This pattern is consistent with blacksmith plovers piercing the eggs, as all of the predators we recorded took the entire clutch. Two additional clutches were taken at night by mongooses (presumed to be water mongoose *Atilax paludinosus*), where nonvisual sensory cues are likely to play more of a role in detection. The videos also revealed one nest being washed away by high water. One additional nest not videoed was presumed to have been depredated by a mongoose, based on the presence of tracks. Monitoring of other plover species at the same field site with motion‐sensitive cameras revealed a similar pattern, with high rates of predation from pied crows (taking one chestnut‐banded plover nest *Charadrius pallidus*, and two blacksmith plover nests) and one instance of predation by a predatory mammal (possibly a black‐backed jackal *Canis mesomelas mesomelas*) of a crowned plover nest *Vanellus coronatus* on nearby farmland.

### 2.2 Photography

**Figure 1:**
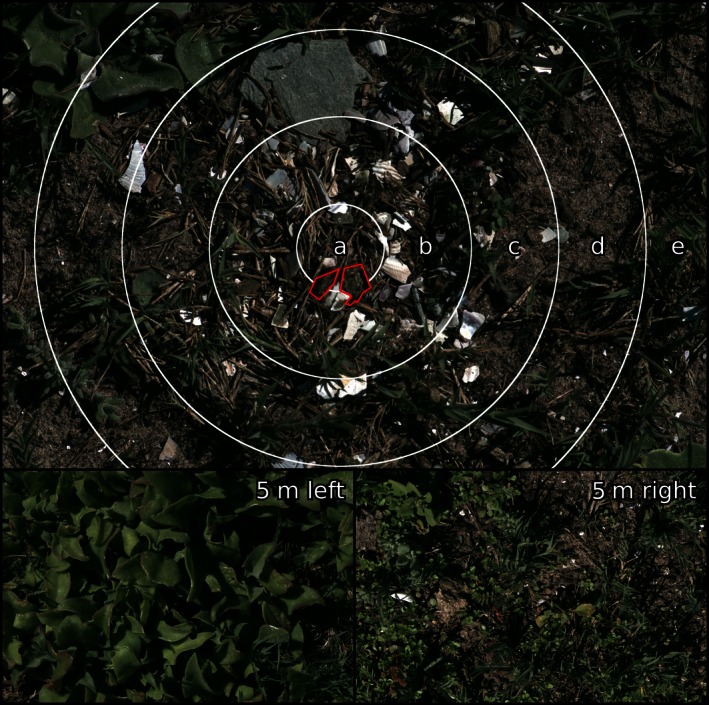
Kittlitz's plovers rarely have clearly defined nest edges; instead, the amount of loose material they move decreases with distance from the nest. We therefore measured the nest visual appearance in concentric rings centered on the nest (white rings, “a” to “e”). Zone “a” was always composed of entirely modified material, while zone “e” was typically minimally modified or unmodified. Any egg visible through the nest‐covering material was excluded from analysis (red). We also took a pair of control photographs 5 m either side of each nest as random background samples that are highly unlikely to have been substantially modified by the plovers (bottom left and right)

Our photography methods followed Troscianko et al. (2016) and Wilson‐Aggarwal, Troscianko, Stevens, and Spottiswoode (2016). Prior to photography, nests were approached at a slow walking pace to allow the Kittlitz' plovers to cover their clutches with nesting materials. Nests were photographed with two Nikon D7000 cameras that had been converted to full‐spectrum sensitivity, fitted with Micro Nikkor AF‐S VR 105‐mm lenses that transmit ultraviolet (UV) light. Photographs were taken through Baader Venus‐U filters (transmitting UV wavelengths from ~320 to 380 nm) and Baader UV/IR cut filters (transmitting human‐visible wavelengths from ~400 to 700 nm). For further details and spectral sensitivities of this camera setup, see Troscianko and Stevens (2015). Photographs were taken from a height of 1.25 m directly above the nest with the nest material in place. Without moving the camera, further images were taken after we pushed the covering nest material to the side of the nest (for details on finding the center of the nest, see below). Two further control photographs were taken approximately 5 m either side of the nest (see Figure 1). This distance was chosen as being representative of the nesting habitat of the plovers, but sufficiently far from the nest to not risk having had its visual appearance substantially altered by the plovers' nest‐covering behavior. Lighting conditions were controlled for and reflectance measured by normalizing the images against 40% reflectance Spectralon (Labsphere) gray standards (Stevens et al., 2007), placed flat on the ground adjacent to the nest or in the center of control photographs. A sequential normalization procedure was used, whereby photographs of the gray standards were taken immediately after the nest and control photographs while ensuring there were no changes in lighting conditions or camera settings (Stevens, Stoddard, & Higham, 2009; Troscianko & Stevens, 2015). All photographs were taken in direct sunlight conditions and not within one hour of sunrise or sunset, to ensure that lighting conditions were as diffuse and as consistent as possible between images (e.g., to remove the light spill or shadows of nearby objects). Eggs were removed from the nests and photographed in diffuse, shade conditions (approximating D65 standard illumination) against a white background and with a gray standard and ball bearing (for use as a scale bar) in the same photograph.

### 2.3 Image analysis

**Figure 2:**
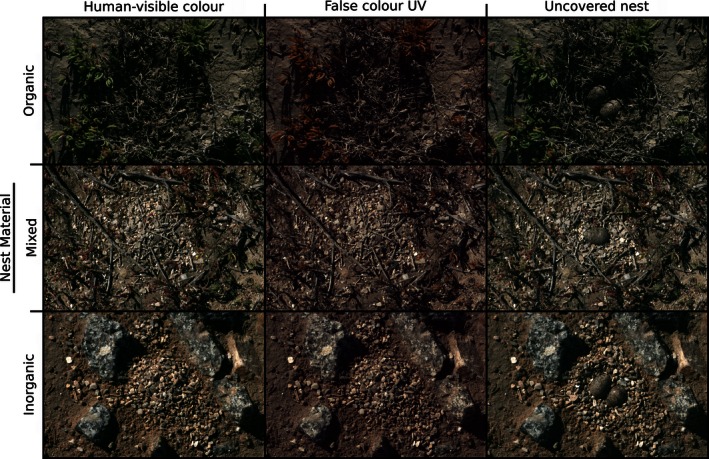
Three sample nest images. Images in the left column show LW, MW, and SW peafowl cone‐catch quanta, the central column shows false color MW, SW, and VS cone‐catch, and the right column shows the uncovered nest. Cone‐catch values were square root transformed for this figure to optimize viewing. The rows show examples of the different nest materials, with organic material (primarily sticks and dried leaves, top row), a mixture (central row), and inorganic material (primarily small stones and sand)

Images were calibrated and analyzed using the multispectral image calibration and analysis toolbox (Troscianko & Stevens, 2015; Troscianko et al., 2016). Camera traps demonstrated that the principal diurnal threats to nest survival were from birds with violet‐sensitive (VS) visual systems (as opposed to UV systems, Ödeen & Håstad, 2013); see Results. Images were therefore converted to the cone‐catch values of the peafowl (*Pavo cristatus*); this is a model visual system often used for VS bird species (Hart, 2002). The center of the nest was manually located in each image from photographs showing the uncovered nest (see Figure 2), and then four concentric rings at radius intervals of 650 pixels (px) were generated around this center, creating five zones in the nest image, labeled from “a” at the center to “e” at the edge of the image (Figure 1). Any egg visible through the nest material was removed from the analysis. The central zone (“a”) always unambiguously comprised nest material or partially visible eggs; however, the degree of nest material manipulation is expected to reduce with distance from the center, resulting in the nests' observed gradual edges (see Figure 2 and Supplementary Video). The use of concentric rings and 5‐m control images in the analysis reflects this spatial shift from unambiguous nest material to unmodified background. Image statistics were generated independently in each ring and using the entirety of the 5‐m control images.

Peafowl double cone responses scaled down uniformly to 10 px/mm were used to generate luminance data, which included mean luminance, contrast (luminance standard deviation divided by mean luminance), and pairwise luminance difference measurements (based on comparing the luminance histograms of the egg and background sample using 100 bins; Troscianko & Stevens, 2015). Pattern processing is thought largely to rely on luminance information (Osorio & Vorobyev, 2005), so was also processed from double cone responses with bandpass analysis at 15 levels, from 2 to 256 px in multiples of √2. Pattern metrics included the dominant pattern size (the spatial frequency with the highest energy), the maximum energy (the energy at the maximum frequency), summed energy (the energy summed across all scales), proportion energy (the maximum energy divided by the summed energy, which describes the diversity of pattern sizes), mean energy, and energy standard deviation (Chiao et al., 2009; Stoddard & Stevens, 2011). In addition, pairwise pattern difference measures were calculated between the clutch and background regions, and zone “a” and all other background regions, describing the level of dissimilarity between any two patterns (Troscianko & Stevens, 2015).

Nesting habitats often contained background objects that had different colors, such as vegetation, rocks, or mud (see Figures 1 and 2). Taking the mean color measurement across these discrete objects would not generate representative color information and might even produce intermediate colors that do not exist in the scene. We therefore used a custom‐written “agglomerative hierarchical clustering algorithm” to separate out these different colors based on peafowl color discrimination, as follows. For color analysis, images were scaled to a uniform 5 px/mm. The code first clustered each pixel with its nearest neighbor based on discrimination units of “just noticeable differences” (JNDs; Vorobyev & Osorio, 1998), calculating new color averages for each clustered group on each pass. Neighbors were joined from larger receptive fields in consecutive passes, starting with a 1 px radius, and doubling the radius with each pass. Analysis was performed after seven passes, which on average split each clutch into 1.86 discrete colors, each concentric background ring into 9.34 colors, and the control images into 42.19 colors. Each of the colors identified in each clutch was then compared to all of the colors in each background zone based on JNDs, and a weighted average JND was created that took into account the percentage area coverage of each color. Therefore, discrete colors that were rare in a scene contributed less to the weighted JND than did more abundant background colors. The same pairwise color differences were also created to compare the colors in zone “a” to all other background zones.

The materials available to Kittlitz' plovers for lining and covering their nests varied between locations, from almost entirely inorganic material (small stones) to almost entirely dried vegetation (dried sticks and leaves). We scored each nest visually for the nest material composition, from 0 (>90% inorganic material), to 1 (a mixture of materials), to 2 (>90% organic material); Figure 2 shows examples of all three scores.

### 2.4 Statistics

Statistics were performed in R version 3.2.2. All pattern metrics and JND values were log‐transformed to produce a normal error distribution. Spearman covariance matrices of camouflage metrics revealed high autocorrelation between numerous pattern variables (values >0.5 or <−0.5; (Zuur, Ieno, Walker, Saveliev, & Smith, 2009)). Therefore, only dominant pattern size and maximum energy were used as pattern descriptive statistics as these were uncorrelated with one another. Survival was modeled in mixed‐effects Cox models (coxme package, version 2.2‐5). Survival time was measured in days. Nests for which the outcome was uncertain, nests which were still intact at the end of the fieldwork, and nests which had hatched were all censored from the survival model at their last recorded time (censoring in survival models allows all survival data to be included until the point of censoring, even if the outcome of any given nest is uncertain). Survival was modeled against camouflage variables, with zone and nest material scores included as ordinal variables, and nest as a random factor because multiple background zones were measured in each nest. Nest appearance as a function of increasing distance from the eggs was tested with cumulative link mixed models (ordinal package, version 2015.6‐28), modeling zone as an ordinal dependent variable against camouflage variables and nest material score, with nest as a random effect. Likelihood ratio tests were used to simplify a full model containing all two‐way interactions, resulting in a final model. Significance levels were generated by dropping single terms from the model while maintaining marginality and using chi‐square tests between models.

## 3 Results

### 3.1 Nest covering

A total of 35 Kittlitz's plover nests were photographed and monitored from 2 August to 14 September 2013. Observations from motion‐triggered video cameras and direct observations on foot and from cars revealed Kittlitz's plovers covering their nests on approach of humans, pied crows, and blacksmith plovers (see Supplementary Video). Large flocks of Hartlaub's gulls *Chroicocephalus hartlaubii* frequently congregated within 20 m of some Kittlitz's plover nests; however, we observed neither nest covering in response to their presence, nor predation by the gulls. This nest‐covering behavior only in the presence of potential threats suggests that one of the roles of nest covering is indeed concealment (Maclean, 1974).

**Figure 3:**
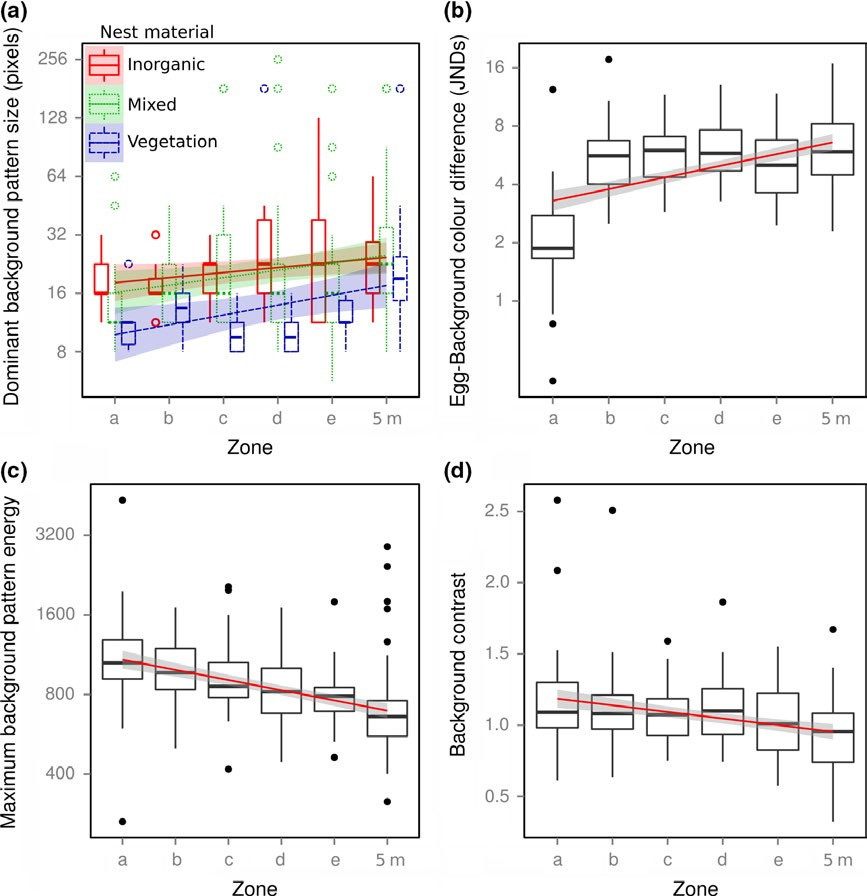
Boxplots showing the camouflage metrics found to vary significantly with distance from the nest. Regression lines also show standard error (shaded region). Zones “a” to “e” are concentric rings increasing in distance from the center of the nest, and the final zones (“5 m”) are control images taken 5 m from each nest

Perfect background matching would predict that there should be no differences in appearance at different distances (zones) from the center of the nest, implying no variables in our model would be significant. Any camouflage variables that do vary consistently between zones suggest that background matching is imperfect; additional interactions with nesting material and the match to the colors and patterns of the eggs themselves test for limitations and trade‐offs in nest appearance. Cumulative link mixed models revealed a number of camouflage variables that varied significantly with distance from the center of the Kittlitz's plover nests, implying that nest covering does not provide perfect background matching. The final model demonstrated an interaction with nesting material score and dominant pattern size (likelihood ratio test (LRT) = 8.72, *p* = .013); this arose because the centers of nests composed of dried vegetation had smaller dominant pattern sizes than nests composed of inorganic materials and were a worse match to the surrounding dominant pattern sizes. Nests covered with inorganic materials were more consistent in their pattern sizes across the zones (i.e., there was a less strong correlation between pattern size and distance from the center of the nest for inorganic material than organic), suggesting they achieved a better background pattern match (Figure 3a). There was also a significant interaction between dominant pattern size and maximum energy (LRT = 13.60, *p* < .001); maximum energy values decreased with distance from the nest, meaning the background patterns were smaller and the dominant patterns had lower contrast further from the nest (Figure 3c). Color difference between the eggs and their backgrounds increased significantly with distance (LRT = 86.28, *p* < .001), indicating the nesting material was a better color match to the eggs than the surrounding background (Figure 3b). Contrast decreased significantly with distance (LRT = 102.11, *p* < .001), meaning the center of the nests had higher contrast than their surrounds (Figure 3d).

### 3.2 Nest survival

Just three nests were known to survive to hatching. Eighteen were depredated (video footage confirmed four of these events), while others were presumed to have been depredated because the eggs disappeared before sufficient incubation time and with no evidence of hatching (methods for determining clutch fate followed Troscianko et al., 2016). Eight nests were still present at the end of our fieldwork, four disappeared without a clear cause, one nest was destroyed by high water, and one was deserted by the parents.

Survival analyses did not reveal any correlations between nest camouflage and likelihood of predation (no models were better than the null). These models included pairwise comparisons between zone “a” and all other background zones, pairwise comparisons between the clutch and all background zones, and overall descriptive statistics across all the background zones.

## 4 Discussion

Kittlitz's plovers conceal their nests by covering them with material that they accumulate in advance, allowing us to determine what visual cues (if any) they use when selecting covering material and how this affected their survival in a natural system. The nests of Kittlitz's plovers rarely have clear boundaries, such that the arranged material looks continuous with the background. We found that the appearance of the nests—as modeled through the visual system of their main predator—changed with distance from the nest center, indicating that nests are not a perfect match to their backgrounds. Moreover, this systematic change in appearance differed between camouflage variables: while the selected materials were a near‐perfect match to the color of the eggs, consistent with Mayer et al. (2009), they created higher contrast and pattern differences with their backgrounds. Such a mismatch might arise from a trade‐off between different aspects of camouflage created by material constraints or thermal considerations, from an intentional camouflage strategy, or from a combination of the two.

The plovers' eggs were a poor match to their overall surrounds, meaning that they would be visible to predators against their backgrounds without nesting materials to cover them (Figure 3b). The plovers could therefore either select covering material that matches the background, thus creating no color cues for predators, or they could select material that matches the color of the eggs, such that any eggs visible through the material do not create a strong color contrast. Alternatively, the plovers might not be selective in their choice of material, in which case we would expect the nest material to be closer in color to the surrounding environment than to the eggs. We found that the nesting material was a near‐perfect color match to the eggs, irrespective of whether they were composed of dried vegetation or stones (median = 1.9 JNDs, see Figure 3b; Siddiqi, Cronin, Loew, Vorobyev, & Summers, 2004). However, these materials were a poor color match to their surrounds, suggesting that in covering their nests, the plovers used material that matched their egg color better than the nest's background color, thereby making the entire nest a worse color match to its surrounds. The gradual decrease in nesting material with distance observed in the Kittlitz's plover nests could mitigate for the imperfect color of the nest. Such a strategy of gradual color shifts could enhance camouflage by confusing the local color constancy mechanisms of the receiver (Hurlbert, 1999), a potential camouflage mechanism that has received almost no attention to date.

In addition to the color‐based trade‐offs above, the Kittlitz's plover nests also varied from their backgrounds in pattern and contrast. Nests had higher contrast and higher maximum pattern energy (the contrast of the dominant pattern scale) than their surroundings. Nests covered with entirely organic material also had smaller pattern details than the average surroundings, demonstrating that organic and inorganic materials do differ substantially in appearance even though the plovers managed to match the colors of their eggs perfectly irrespective of material. Such systematic changes from a background‐matching strategy could result from two mechanisms that are not mutually exclusive: limitations in material availability and an adaptive camouflage strategy. The material limitation hypothesis makes the assumption that the available nesting materials are unable to match all of the desired appearance characteristics simultaneously; for example, if the plovers were selecting materials for a perfect color match with their eggs, these same materials may be unable to match the egg or background appearance characteristics closely. If this is the case, our findings suggest that the plovers value color matching above other camouflage variables, contrary to our predictions that pattern and contrast should be most important (Troscianko et al., 2016; Wilson‐Aggarwal et al., 2016). Alternatively, the adaptive strategy hypothesis would suggest that the nests' deviation from perfect background matching reflects a shift in camouflage strategy from background matching to some other (presumably more effective) strategy. The most well‐documented alternative to background matching is disruptive coloration, where the prey's edges are broken up by high‐contrasting patches (Thayer 1909; Cott 1940; Cuthill et al., 2005). The higher luminance contrasts and higher pattern contrasts of the plover nests compared to the background are consistent with such a disruptive coloration strategy (Troscianko, Lown, Hughes, & Stevens, 2013). Although the Kittlitz's plover nests generally have no clear boundaries or fixed shapes, a recent study has demonstrated the effectiveness of disruptive camouflage in prey with graduated boundaries (Webster, Godin, & Sherratt, 2015), suggesting that the higher contrast of the nests could help to disrupt the predator's perception of a nest shape.

We found no evidence that camouflage affected the likelihood of clutches surviving to hatching in this study. This could be due to a modest sample size of 35 nests, which is comparatively small for survival analysis, although predation rates were extremely high in this system. Predation of poorly camouflaged nests could also have been so high that the frequency with which we monitored nests was insufficient to detect any effect of camouflage metrics. Alternatively, the lack of any detected effect of camouflage on survival could indicate that the primary purpose of nest covering is something other than concealment, such as thermal insulation, or it could indicate that the predators are utilizing some other cue to find nests, such as olfactory cues or watching the adult plovers for their covering scuffle. However, we suggest that the primary function of nest covering at our field site is likely to be visual concealment, for the following reasons. First, nest‐covering behavior was specifically associated with the presence of potential threats, but not the presence of animals that did not pose a threat. Second, a number of other plover species nest in the same habitat at the same time and lay similarly sized eggs without requiring further nest insulation (e.g., chestnut‐banded plovers, three‐banded plovers *C. tricollaris*, and white‐fronted plovers *C. marginatus*). Third, artificial Kittlitz's plover nests at our field site in a subsequent breeding season were more likely to be detected by predators than were uncovered nests (Ferguson, 2016). However, given the large geographical distribution of Kittlitz's plovers throughout much of Africa, there are likely to be sites where thermal factors are more important, and this may have influenced the evolution of the behavior in the species as a whole.

Our study highlighted the importance of the pied crow as a nest predator, not just for the Kittlitz's plover, but also for other plover species monitored at this field site. Pied Crows have greatly increased in numbers in our study region over the last two decades (Cunningham, Madden, Barnard, & Amar, 2016). We were unable to determine how many individual pied crows were responsible for the predation events in this study, but they were most often observed in pairs, although we occasionally observed larger flocks at one of the study sites that was adjacent to a farm. It is therefore possible that the majority of Kittlitz's plover predation events were caused by a single individual or pair at each site. If so, then these intelligent, visually guided predators would be afforded substantial learning opportunities, allowing them to specialize in finding the concealed Kittlitz's plover nests using a search image (Bond & Kamil, 1999). The use of high‐contrast nest‐covering material we observed could also be a salient factor that enhances learning rates, as experiments on humans show that while high‐contrast prey benefit from increased disruptive camouflage, people also learnt to find higher contrast prey faster over successive prey encounters (Troscianko et al., 2013).

**Figure 4:**
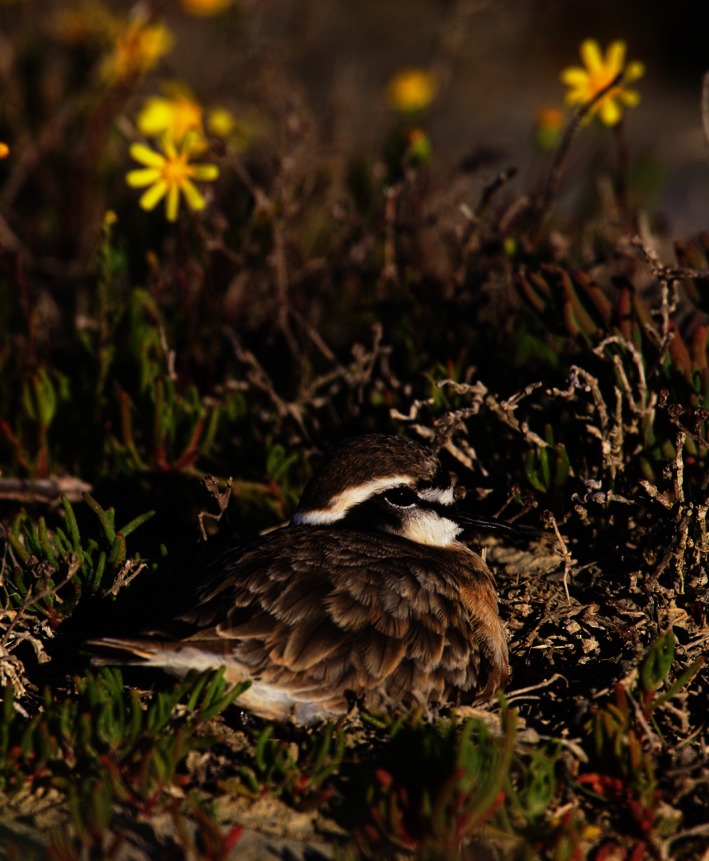
Kittlitz's plover sitting on its nest. When the plover sees a threat approaching, it will perform a “leaving‐scuffle” over the nest to conceal its eggs. The plovers carefully select materials for covering their nests in advance that are a near‐perfect match to the color of their eggs when viewed by predators

The appearance of most camouflaged animals represents a compromise between the different traits that best protect them from detection in range of habitat types and visual backgrounds where they are vulnerable to predation (Endler, 1978) and can also be constrained by their thermal properties (Amat et al., 2012; Gómez et al., 2015; Grant, 1982; Mayer et al., 2009; Wilson‐Aggarwal et al., 2016). The nest‐covering behavior of Kittlitz's plovers therefore provides a useful study system for investigating camouflage that is not subject to the same visual constraints as the adults (fig. 4) and eggs, in that it can be plastically modified within and between breeding attempts. Surprisingly, our data suggest that Kittlitz's plovers did not select nest material to match their specific nest background, but rather to match the color of their eggs. However, the selection criteria used by the plovers when collecting nesting material—and how exactly the color match is achieved—remain unknown. Future work should investigate whether our findings reflect a trade‐off between selecting materials that matched egg color at the expense of rendering the covered nest a poor pattern and contrast match to the background, or whether the systematic use of materials with high‐contrast and small‐pattern grain size was adaptive, reflecting a switch from a background‐matching strategy to a disruptive coloration strategy. Our study also suggests that increasing pied crow numbers (Cunningham et al., 2016) could pose a threat if high‐contrast nests offer a salient learning cue to these predators. If so, then changes in predator communities and abundance may have significant effects on the adaptive value of antipredator strategies such as camouflage.

## Data Accessibility

Data are available in the Open Research Exeter repository available here: http://hdl.handle.net/10871/23232. The supplementary video is available here: https://youtu.be/QI1zjdQnjlU.

## Conflict of Interest

None declared.

## References

[ece32494-bib-0001] Amat, J. A. , Monsa, R. , & Masero, J. A. (2012). Dual function of egg‐covering in the Kentish plover *Charadrius alexandrinus* . Behaviour, 149, 881–895.

[ece32494-bib-0002] Bailey, I. E. , Muth, F. , Morgan, K. , Meddle, S. L. , & Healy, S. D. (2014). Birds build camouflaged nests. The Auk, 132, 11–15.

[ece32494-bib-0003] Bond, A. B. , & Kamil, A. C. (1999). Searching image in blue jays: Facilitation and interference in sequential priming. Animal Learning & Behavior, 27, 461–471.

[ece32494-bib-0004] Castilla, A. M. , Dhondt, A. A. , Díaz‐Uriarte, R. , & Westmoreland, D. (2007). Predation in ground‐nesting birds: An experimental study using natural egg‐color variation. Avian Conservation and Ecology‐Écologie et conservation des oiseaux, 2, 2.

[ece32494-bib-0005] Chiao, C.‐C. , Chubb, C. , Buresch, K. , Siemann, L. , & Hanlon, R. T. (2009). The scaling effects of substrate texture on camouflage patterning in cuttlefish. Vision Research, 49, 1647–1656. 19362570 10.1016/j.visres.2009.04.002

[ece32494-bib-0006] Colwell, M. A. , Meyer, J. J. , Hardy, M. A. , Mcallister, S. E. , Transou, A. N. , Levalley, R. R. , & Dinsmore, S. J. (2011). Western Snowy Plovers *Charadrius alexandrinus nivosus* select nesting substrates that enhance egg crypsis and improve nest survival. Ibis, 153, 303–311.

[ece32494-bib-0500] Cott, H.B . (1940) Adaptive Coloration in Animals. Oxford University Press.

[ece32494-bib-0007] Cunningham, S. J. , Madden, C. F. , Barnard, P. , & Amar, A. (2016). Electric crows: Powerlines, climate change and the emergence of a native invader. Diversity and Distributions, 22, 17–29.

[ece32494-bib-0008] Cuthill, I. C. , Stevens, M. , Sheppard, J. , Maddocks, T. , Párraga, C. A. , & Troscianko, T. S. (2005). Disruptive coloration and background pattern matching. Nature, 434, 72–74. 15744301 10.1038/nature03312

[ece32494-bib-0009] Endler, J. A. (1978). A predator's view of animal color patterns In Hecht M. K., Steere W. C., & Wallace B. (Eds.), Evolutionary biology, evolutionary biology (pp. 319–364). US: Springer.

[ece32494-bib-0010] Fast, P. L. , Gilchrist, H. G. , & Clark, R. G. (2010). Nest‐site materials affect nest‐bowl use by Common Eiders (*Somateria mollissima*). Canadian Journal of Zoology, 88, 214–218.

[ece32494-bib-0011] Ferguson, A. (2016) Using conditioned food aversion (CFA) to reduce pied crow (Corvus albus) predation of plover nests. Master's Thesis, DST/NRF Centre of Excellence, Percy FitzPatrick Institute of African Ornithology, University of Cape Town.

[ece32494-bib-0012] Gómez, J. , Pereira, A. I. , Pérez‐Hurtado, A. , Castro, M. , Ramo, C. , & Amat, J. A. (2015). A trade‐off between overheating and camouflage on shorebird eggshell colouration. Journal of Avian Biology, 46, 1–8.

[ece32494-bib-0013] Grant, G. S. (1982). Avian incubation: Egg temperature, nest humidity, and behavioral thermoregulation in a hot environment. Ornithological Monographs, 30, 1–75.

[ece32494-bib-0014] Hall, K. R. L. (1958). Observations on the nesting sites and nesting behaviour of the Kittlitz's Sandplover *Charadrius pecuarius* . Ostrich, 29, 113–125.

[ece32494-bib-0015] Hall, K. R. L. (1960). Egg‐covering by the white‐fronted Sandplover *Charadrius marginatus* . Ibis, 102, 545–553.

[ece32494-bib-0016] Hansell, M. H. (1996). The function of lichen flakes and white spider cocoons on the outer surface of birds' nests. Journal of Natural History, 30, 303–311.

[ece32494-bib-0017] Hart, N. S. (2002). Vision in the peafowl (Aves: *Pavo cristatus*). Journal of Experimental Biology, 205, 3925–3935. 12432014 10.1242/jeb.205.24.3925

[ece32494-bib-0018] Hilton, G. M. , Hansell, M. H. , Ruxton, G. D. , Reid, J. M. , Monaghan, P. , & Brittingham, M. (2004). Using artificial nests to test importance of nesting material and nest shelter for incubation energetics. The Auk, 121, 777–787.

[ece32494-bib-0019] Hultgren, K. M. , & Stachowicz, J. J. (2009). Evolution of decoration in majoid crabs: A comparative phylogenetic analysis of the role of body size and alternative defensive strategies. The American Naturalist, 173, 566–578. 10.1086/597797 19278336

[ece32494-bib-0020] Hurlbert, A. (1999). Colour vision: Is colour constancy real? Current Biology, 9, R558–R561. 10469555 10.1016/s0960-9822(99)80354-6

[ece32494-bib-0021] Kang, C.‐K. , Moon, J.‐Y. , Lee, S. , & Jablonski, P. G. (2013). Moths on tree trunks seek out more cryptic positions when their current crypticity is low. Animal Behaviour, 86, 587–594.

[ece32494-bib-0022] Kang, C. , Moon, J.‐Y. , Lee, S.‐I. , & Jablonski, P. G. (2014a). Moths use multimodal sensory information to adopt adaptive resting orientations. Biological Journal of the Linnean Society, 111, 900–904.

[ece32494-bib-0023] Kang, C. , Stevens, M. , Moon, J. , Lee, S.‐I. , & Jablonski, P. G. (2014b). Camouflage through behavior in moths: The role of background matching and disruptive coloration. Behavioral Ecology, 26, 45–54.

[ece32494-bib-0024] Kreisinger, J. , & Albrecht, T. (2008). Nest protection in mallards *Anas platyrhynchos*: Untangling the role of crypsis and parental behaviour. Functional Ecology, 22, 872–879.

[ece32494-bib-0025] Lee, B. Y. , Parra‐Velandia, F. J. , Ng, N. K. , & Todd, P. A. (2014). An unusual form of camouflage in the mangrove crab *Clistocoeloma merguiense* . Bulletin of Marine Science, 90, 967–968.

[ece32494-bib-0026] Lovell, P. G. , Ruxton, G. D. , Langridge, K. V. , & Spencer, K. A. (2013). Egg‐laying substrate selection for optimal camouflage by quail. Current Biology: CB, 23, 260–264. 23333313 10.1016/j.cub.2012.12.031

[ece32494-bib-0027] Maclean, G. L. (1974). Egg‐covering in the Charadrii. Ostrich, 45, 167–174.

[ece32494-bib-0028] Marshall, K. L. , Philpot, K. E. , & Stevens, M. (2016). Microhabitat choice in island lizards enhances camouflage against avian predators. Scientific Reports, 6, 19815. 26804463 10.1038/srep19815 PMC4726299

[ece32494-bib-0029] Mayani‐Parás, F. , Kilner, R. M. , Stoddard, M. C. , Rodríguez, C. , & Drummond, H. (2015). Behaviorally induced camouflage: A new mechanism of avian egg protection. The American Naturalist, 186, E91–E97. 10.1086/682579 26655580

[ece32494-bib-0030] Mayer, P. M. , Smith, L. M. , Ford, R. G. , Watterson, D. C. , McCutchen, M. D. , & Ryan, M. R. (2009). Nest construction by a ground‐nesting bird represents a potential trade‐off between egg crypticity and thermoregulation. Oecologia, 159, 893–901. 19145449 10.1007/s00442-008-1266-9

[ece32494-bib-0031] Merilaita, S. , & Dimitrova, M. (2014). Accuracy of background matching and prey detection: Predation by blue tits indicates intense selection for highly matching prey colour pattern (ed S Lewis). Functional Ecology, 28, 1208–1215.

[ece32494-bib-0032] Ödeen, A. , & Håstad, O. (2013). The phylogenetic distribution of ultraviolet sensitivity in birds. BMC Evolutionary Biology, 13, 36. 23394614 10.1186/1471-2148-13-36 PMC3637589

[ece32494-bib-0033] Opermanis, O. (2004). Appearance and vulnerability of artificial duck nests to avian predators. Journal of Avian Biology, 35, 410–415.

[ece32494-bib-0034] Osorio, D. , & Vorobyev, M. (2005). Photoreceptor sectral sensitivities in terrestrial animals: Adaptations for luminance and colour vision. Proceedings of the Royal Society B: Biological Sciences, 272, 1745–1752. 16096084 10.1098/rspb.2005.3156 PMC1559864

[ece32494-bib-0035] Prokop, P. , & Trnka, A. (2010). Why do grebes cover their nests? Laboratory and field tests of two alternative hypotheses. Journal of Ethology, 29, 17–22.

[ece32494-bib-0036] Reid, J. M. , Cresswell, W. , Holt, S. , Mellanby, R. J. , Whitfield, D. P. , & Ruxton, G. D. (2002). Nest scrape design and clutch heat loss in Pectoral Sandpipers (*Calidris melanotos*). Functional Ecology, 16, 305–312.

[ece32494-bib-0037] Ruxton, G. D. , & Stevens, M. (2015). The evolutionary ecology of decorating behaviour. Biology Letters, 11, 2015.0325. 10.1098/rsbl.2015.0325 PMC4528480 26041868

[ece32494-bib-0038] Schaefer, H. M. , & Stobbe, N. (2006). Disruptive coloration provides camouflage independent of background matching. Proceedings of the Royal Society of London B: Biological Sciences, 273, 2427–2432. 10.1098/rspb.2006.3615 PMC1634905 16959631

[ece32494-bib-0039] Siddiqi, A. , Cronin, T. W. , Loew, E. R. , Vorobyev, M. , & Summers, K. (2004). Interspecific and intraspecific views of color signals in the strawberry poison frog *Dendrobates pumilio* . Journal of Experimental Biology, 207, 2471–2485. 15184519 10.1242/jeb.01047

[ece32494-bib-0040] Skelhorn, J. , Rowland, H. M. , Speed, M. P. , & Ruxton, G. D. (2010). Masquerade: Camouflage without crypsis. Science, 327, 51–51. 20044568 10.1126/science.1181931

[ece32494-bib-0041] Solis, J. C. , & De Lope, F. (1995). Nest and egg crypsis in the ground‐nesting stone curlew *Burhinus oedicnemus* . Journal of Avian Biology, 26, 135–138.

[ece32494-bib-0501] Stevens, M ., Parraga, C.A ., Cuthill, I.C ., Partridge, J.C. , & Troscianko, T.S . (2007) Using digital photography to study animal coloration. Biological Journal of the Linnean Society, 90, 211–237.

[ece32494-bib-0042] Stevens, M. , Stoddard, M. C. , & Higham, J. P. (2009). Studying primate color: Towards visual system‐dependent methods. International Journal of Primatology, 30, 893–917.

[ece32494-bib-0043] Stoddard, M. C. , & Stevens, M. (2011). Avian vision and the evolution of egg color mimicry in the common cuckoo. Evolution, 65, 2004–2013. 21729055 10.1111/j.1558-5646.2011.01262.x

[ece32494-bib-0044] Strassmann, J. E. , Hughes, C. R. , & Queller, D. C. (1990). Colony defense in the social wasp, *Parachartergus colobopterus* . Biotropica, 22, 324–327.

[ece32494-bib-0045] Summers, R. W. , & Hockey, P. A. R. (1981). Egg‐covering behaviour of the white‐fronted plover *Charadrius marginatus* . Ornis Scandinavica, 12, 240–243.

[ece32494-bib-0502] Thayer, A.H . (1909) Concealing‐Coloration in the Animal Kingdom. Macmillan Company.

[ece32494-bib-0046] Troscianko, J. , Lown, A. E. , Hughes, A. E. , & Stevens, M. (2013). Defeating crypsis: Detection and learning of camouflage strategies. PLoS ONE, 8, e73733. 24040046 10.1371/journal.pone.0073733 PMC3769369

[ece32494-bib-0047] Troscianko, J. , & Stevens, M. (2015). Image calibration and analysis toolbox—a free software suite for objectively measuring reflectance, colour and pattern. Methods in Ecology and Evolution, 6, 1320–1331. 27076902 10.1111/2041-210X.12439 PMC4791150

[ece32494-bib-0048] Troscianko, J. , Wilson‐Aggarwal, J. , Stevens, M. , & Spottiswoode, C. N. (2016). Camouflage predicts survival in ground‐nesting birds. Scientific Reports, 6, 19966. 26822039 10.1038/srep19966 PMC4731810

[ece32494-bib-0049] Tyrie, E. K. , Hanlon, R. T. , Siemann, L. A. , & Uyarra, M. C. (2015). Coral reef flounders, *Bothus lunatus*, choose substrates on which they can achieve camouflage with their limited body pattern repertoire. Biological Journal of the Linnean Society, 114, 629–638.

[ece32494-bib-0050] Vorobyev, M. , & Osorio, D. (1998). Receptor noise as a determinant of colour thresholds. Proceedings of the Royal Society of London, Series B: Biological Sciences, 265, 351–358. 9523436 10.1098/rspb.1998.0302 PMC1688899

[ece32494-bib-0051] Wallace, A. R. (1867). Mimicry and other protective resemblances among animals. Westminster Review, London ed. London.

[ece32494-bib-0052] Webster, R. J. , Godin, J.‐G. J. , & Sherratt, T. N. (2015). The role of body shape and edge characteristics on the concealment afforded by potentially disruptive marking. Animal Behaviour, 104, 197–202.

[ece32494-bib-0053] Webster, R. J. , Hassall, C. , Herdman, C. M. , Godin, J.‐G. J. , & Sherratt, T. N. (2013). Disruptive camouflage impairs object recognition. Biology Letters, 9, 20130501. 24152693 10.1098/rsbl.2013.0501 PMC3871342

[ece32494-bib-0054] Wilson‐Aggarwal, J. , Troscianko, J. , Stevens, M. , & Spottiswoode, C. N. (2016). Escape distance in ground‐nesting birds differs with individual level of camouflage. The American Naturalist, 188, 231–239. 10.1086/687254 27420787

[ece32494-bib-0055] World Weather Online . (2016) Langebaanweg, South Africa Weather Averages | Monthly Average High and Low Temperature | Average Precipitation and Rainfall days | World Weather Online. URL http://www.worldweatheronline.com/langebaanweg-weather-averages/western-cape/za.aspx. Accessed 6 May 2016.

[ece32494-bib-0056] Zuur, A. , Ieno, E. N. , Walker, N. , Saveliev, A. A. , & Smith, G. M. (2009). Mixed effects models and extensions in ecology with R. New York: Springer.

